# A Self‐Reconstructed Bifunctional Electrocatalyst of Pseudo‐Amorphous Nickel Carbide @ Iron Oxide Network for Seawater Splitting

**DOI:** 10.1002/advs.202200146

**Published:** 2022-03-25

**Authors:** Hao Zhang, Songyuan Geng, Mengzheng Ouyang, Hossein Yadegari, Fang Xie, D. Jason Riley

**Affiliations:** ^1^ Department of Materials and London Center for Nanotechnology Imperial College London London SW7 2AZ UK; ^2^ Department of Chemistry Imperial College London London SW7 2AZ UK; ^3^ Department of Earth Science and Engineering Imperial College London London SW7 2AZ UK

**Keywords:** iron oxide, lattice oxygen, nickel carbide, seawater splitting, self‐reconstruction

## Abstract

Here, a sol‐gel method is used to prepare a Prussian blue analogue (NiFe‐PBA) precursor with a 2D network, which is further annealed to an Fe_3_O_4_/NiC_x_ composite (NiFe‐PBA‐gel‐cal), inheriting the ultrahigh specific surface area of the parent structure. When the composite is used as both anode and cathode catalyst for overall water splitting, it requires low voltages of 1.57 and 1.66 V to provide a current density of 100 mA cm^−2^ in alkaline freshwater and simulated seawater, respectively, exhibiting no obvious attenuation over a 50 h test. Operando Raman spectroscopy and X‐ray photoelectron spectroscopy indicate that NiOOH_2–x_ active species containing high‐valence Ni^3+^/Ni^4+^ are in situ generated from NiC_x_ during the water oxidation. Density functional theory calculations combined with ligand field theory reveal that the role of high valence states of Ni is to trigger the production of localized O *2p* electron holes, acting as electrophilic centers for the activation of redox reactions for oxygen evolution reaction. After hydrogen evolution reaction, a series of ex situ and in situ investigations indicate the reduction from Fe^3+^ to Fe^2+^ and the evolution of Ni(OH)_2_ are the origin of the high activity.

## Graphical abstract

An Fe_3_O_4_/NiC_x_ composite (NiFe‐PBA‐gel‐cal) with ultrahigh specific surface area was prepared, and it exhibits excellent seawater splitting performance. The in situ generated NiOOH_2–x_ trigger the production of O *2p* electron holes for the acceleration of oxygen evolution reaction, and the reduction from Fe^3+^ to Fe^2+^ and the evolution of Ni(OH)_2_ are the origin of high activities of hydrogen evolution reaction.

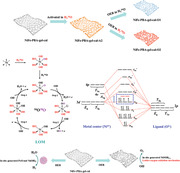

## 1 Introduction

Hydrogen is considered the most promising clean energy fuel owing to its high specific energy (142 MJ kg^–1^) and pollution‐free products.^[^ ^1^, ^2^ ^]^ Electricity‐driven water splitting is currently the most effective way to produce hydrogen.^[^ ^3^, ^4^, ^5^ ^]^ Seawater is the most abundant aqueous electrolyte feedstock on the planet. Compared to purified water splitting, direct seawater electrolysis is a “hits two birds with one stone” technology, it can be used for simultaneous hydrogen production and seawater desalination.^[^ ^6^, ^7^ ^]^ However, to achieve this technology, a highly active and robust oxygen evolution reaction (OER) catalyst, which can maintain seawater electrolysis without driving the chlorine evolution reaction (CER), hypochlorite formation (Equation (1)), or chloride corrosion (Equations (2)–(4)) must be identified^[^ ^8^, ^9^, ^10^ ^]^

(1) $$\;Cl^{-}+2OH^{-}\to \mathrm{Cl}O^{-}+H_{2}O+2e^{-}$$

(2) $$\;M+Cl^{-}\to \mathrm{MC}l_{\mathrm{ads}}+e^{-}$$

(3) $$\mathrm{MC}l_{\mathrm{ads}}+\left(x-1\right)Cl^{-}\to \mathrm{MC}{l_{x}}^{-}+\left(x-2\right)e^{-}$$

(4) $$\mathrm{MC}{l_{x}}^{-}+xOH^{-}\to M{\left(\mathrm{OH}\right)}_{x}+xCl^{-}+e^{-}$$

In the pH range 7.5–14, the standard potential difference between hypochlorite formation and OER is 480 mV.^[^ ^11^ ^]^ The development of electrocatalysts which give high current densities at overpotentials less than 480 mV during alkaline seawater electrolysis is an effective method to inhibit anodic chloride oxidation and hypochlorite formation.^[^ ^12^ ^]^ The formation of a protective layer on the surface of the active sites of the anode material has also been found to be efficient for selective OER over CER and the prevention of chloride corrosion.^[^ ^13^, ^14^ ^]^

To date, substantial activity has been devoted to the development of highly efficient seawater catalysts using transition metal oxides (TMOs),^[^ ^15^, ^16^ ^]^ (hydroxy)hydroxides,^[^ ^17^, ^18^ ^]^ phosphides,^[^ ^19^ ^]^ nitrides,^[^ ^20^ ^]^ and selenides.^[^ ^21^ ^]^ Among these, Fe_3_O_4_ materials have received the most widespread attention due to their diverse crystal structures, abundant reserves, environmental friendliness, and high catalytic activity.^[^ ^22^ ^]^ However, their long‐term stability is an issue as direct exposure to seawater results in chloride‐driven corrosion during electrolysis. Transition metal carbides (TMCs) have significant catalytic activity and offer structural stability.^[^ ^23^, ^24^ ^]^ The wrapping of TMCs on the surface of TMOs can effectively solve the chloride corrosion problem and improve the stability of the catalyst.^[^ ^25^ ^]^ However, TMCs have been found to undergo surface reconstruction in the aqueous and strongly oxidative environments of OER, which makes it more complicated to determine the active sites.^[^ ^26^, ^27^ ^]^ In addition, due to limitations of the synthetic methods, it is difficult to obtain a composite that contains oxides and carbides at the same time, and most carbides have a dense structure, resulting in a significant decrease in mass transfer and active site density.^[^ ^28^ ^]^ Therefore, the development of new synthetic methods is the key to solving this bottleneck.

Sol–gel is a classical wet chemical synthesis method that uses the hydrolysis and condensation of precursors to form a stable sol system which can be slowly polymerized and aged to form a structural gel network.^[^ ^29^ ^]^ Prussian blue analogs (PBAs) are coordination frameworks with the advantages of adjustable cation composition and rich pore structure, which are good precursors for the preparation of TMOs and TMCs once calcined in appropriate atmospheres.^[^ ^30^ ^]^ Most preparations of PBAs yield cubic crystalline structures, in which the exposed {100} surfaces are catalytically inert.^[^ ^31^, ^32^ ^]^ PBAs with a porous 2D network structure would possess more active sites and increased specific surface areas, greatly improving their catalytic performance.

Herein, a sol–gel method is used for the preparation of NiFe‐PBA with 2D cross‐linked networks on a large scale. Sodium citrate is used as a stabilizer and chelating agent for the formation of networks with metal ions, and the inorganic species evenly dispersed in the network. The NiFe‐PBA‐gel precursor was calcined to produce NiFe‐PBA‐gel‐cal (a Fe_3_O_4_/NiC*_x_* composite) inheriting the ultrahigh specific surface area of the parent structure. The as‐prepared catalyst exhibits excellent overall water‐splitting performance in both alkaline freshwater and simulated seawater.

Based on the post‐mortem characterizations and density functional theory (DFT) simulations, the excellent catalytic performance of NiFe‐PBA‐gel‐cal results from self‐reconstruction under oxidation and reduction potentials. After the OER test, the original dispersed Fe_3_O_4_ particles adopted a core–shell structure which exhibited more stable electrochemical performance. Operando Raman spectroscopy and X‐ray photoelectron spectroscopy (XPS) indicated that high‐valence Ni‐containing NiOOH_2−_ *_x_* active species were produced from NiC*_x_*, which is due to the surface reconstruction caused by the metastability of the pseudo‐amorphous structure. DFT calculations combined with ligand field theory (LFT) revealed that high valence states of Ni cause the production of local O 2p electron holes, serving as electrophilic centers for the following water oxidation reaction. In situ ^18^O isotope labeling and the introduction of TMA^+^ as a probe demonstrated that the high OER performance of NiFe‐PBA‐gel‐cal follows the lattice oxygen oxidation mechanism (LOM), which is caused by high‐valence nickel cations and abundant oxygen vacancies. After the hydrogen evolution reaction (HER) measurement, high‐resolution transmission electron microscopy (HRTEM), X‐ray diffraction (XRD), XPS, and Operando Raman analysis were combined to demonstrate the reduction from Fe^3+^ to Fe^2+^ in Fe_3_O_4_ and the evolution from NiC*_x_* to Ni(OH)_2_ leads to the high HER catalytic performance of NiFe‐PBA‐gel‐cal.

## 2 Results and Discussions

### 2.1 Preparation and Characterization of Catalytic Systems

**Figure 1:**
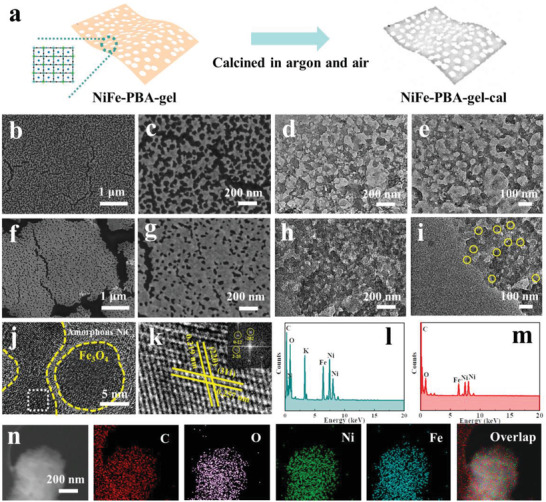
Structure characterization. a) Schematic diagram of the synthesis of NiFe‐PBA‐gel‐cal. b,c) SEM and d,e) TEM images of NiFe‐PBA‐gel. f,g) SEM and h,i) TEM images of NiFe‐PBA‐gel‐cal. j) HRTEM image of NiFe‐PBA‐gel‐cal. k) HRTEM image of the white‐dotted square area in (j) (inset: SAED pattern). l,m) STEM‐EDS of NiFe‐PBA‐gel and NiFe‐PBA‐gel‐cal. n) High‐angle annular dark‐field‐STEM image and EDS‐mapping images of NiFe‐PBA‐gel‐cal.

A two‐step procedure was used to prepare the TMO/TMC composite electrocatalyst precursor, as shown in **Figure** **1**a. A sol–gel method was used for the preparation of a 2D PBA gel network (NiFe‐PBA‐gel), sodium citrate served as a chelating agent to coordinate with metal ions for the formation of a porous network. FeFe‐PBA‐gel was prepared as a comparison containing no Ni element by using iron(II) sulfate heptahydrate in place of nickel(II) sulfate hexahydrate. From the scanning electron microscope (SEM) and TEM images (Figure 1b–e and Figure S1, Supporting Information), it is seen the diameter of the pores is about 30–50 nm and they are intertwined. The NiFe‐PBA‐gel precursor was then annealed in argon and air sequentially to form NiFe‐PBA‐gel‐cal, inheriting the ultra‐large 2D porous network structure (Figure 1f–i), in which the pores are significantly shrunken, less than 20 nm. From the HRTEM images of the calcined composite (Figure 1j,k and Figure S2, Supporting Information), it is noted that the NiC*_x_* contains both amorphous and crystalline phases, and the lattice spacing of 0.303 nm in the crystalline phase corresponds to the (111) plane of NiC*_x_*. It has been reported that transition metal compounds exhibiting amorphous or poorly crystalline properties are more active than their crystalline counterparts.^[^ ^30^, ^33^, ^34^ ^]^ The Fe_3_O_4_ crystals are uniformly distributed on the NiC*_x_* pseudo‐amorphous substrates, and the lattice spacing of 0.219 nm and 0.257 mm corresponds to the (200) and (311) planes of Fe_3_O_4_. Scanning transmission electron microscopy‐energy dispersive X‐ray spectroscopy (STEM‐EDS; Figure 1l,m) and STEM‐mapping (Figure 1n) of NiFe‐PBA‐gel‐cal shows a uniform distribution of C, O, Ni, and Fe across the nanosheets, demonstrating the formation of Fe_3_O_4_ on the pseudo‐amorphous NiC*_x_*. Based on the EDS result, the molar ratio of Ni/Fe is estimated to be ≈3/2, which is consistent with the feed ratio. Compared with the precursor before calcination, N is greatly reduced, which may be because it is oxidized and decomposed at high temperature.

**Figure 2:**
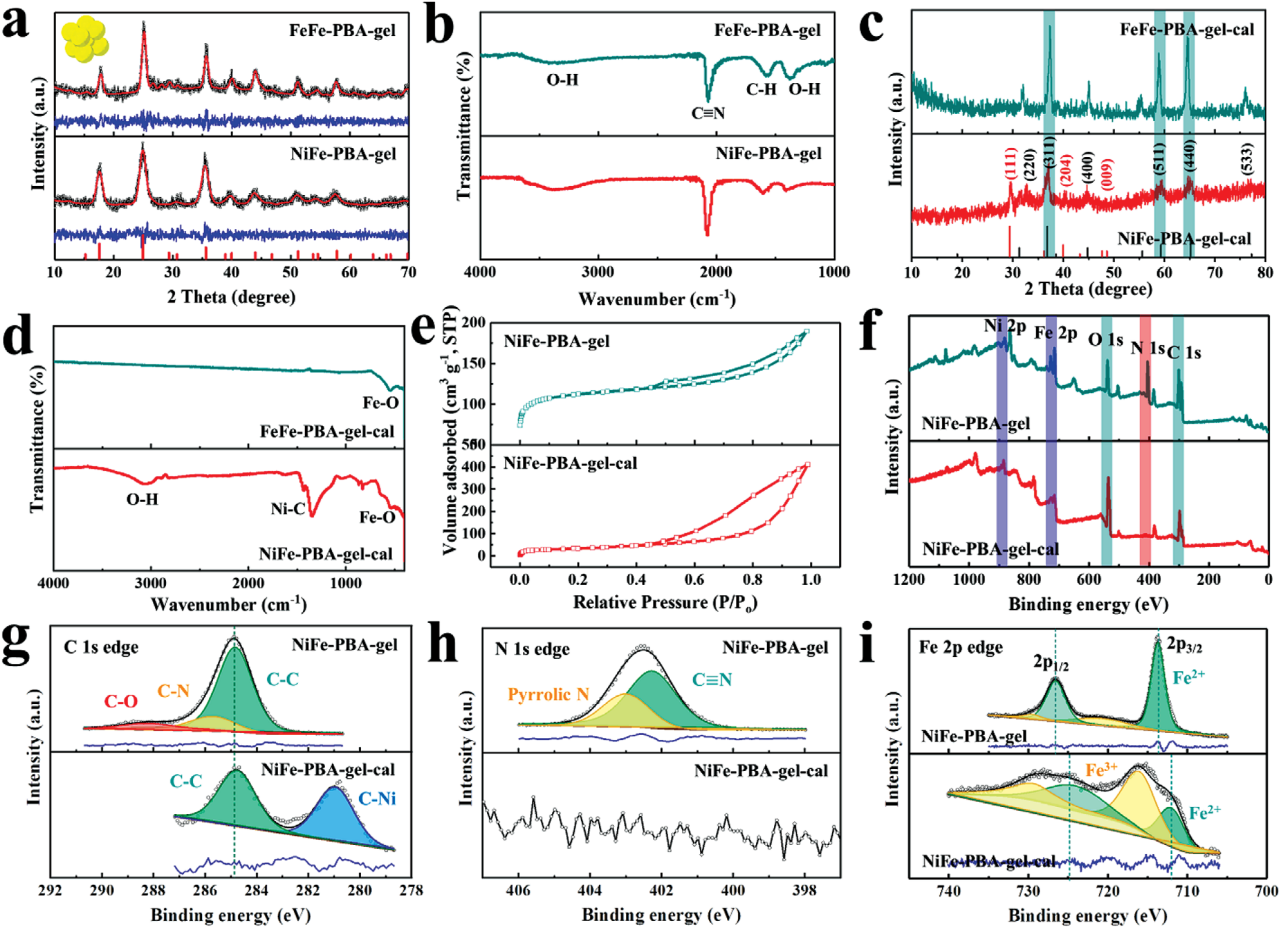
Chemical composition analysis. a) XRD patterns of FeFe‐PBA‐gel and NiFe‐PBA‐gel (inset: unit cell of K_2_NiFe(CN)_6_, black lines: observed patterns, red lines: simulated patterns, blue lines: difference). b) FTIR spectra of FeFe‐PBA‐gel and NiFe‐PBA‐gel. c) XRD patterns of NiFe‐PBA‐gel‐cal. d) FTIR spectra of FeFe‐PBA‐gel‐cal and NiFe‐PBA‐gel‐cal. e) N_2_ adsorption/desorption isotherms of NiFe‐PBA‐gel and NiFe‐PBA‐gel‐cal. f) XPS survey and high‐resolution spectra at g) C 1s edge, h) N 1s edge, and i) Fe 2p edge of NiFe‐PBA‐gel (top) and NiFe‐PBA‐gel‐cal (bottom). The spectra were shown with raw data and fitting data derived by Lorentz–Gaussian function.

XRD patterns from NiFe‐PBA‐gel are displayed in **Figure** **2**a, these match well with simulated K_2_NiFe(CN)_6_ (JCPDS no: 54–0964). FeFe‐PBA‐gel has a slight high‐angle shift, this is because the radius of Fe (124.1 pm) is smaller than that of Ni (124.6 pm), and the replacement of Fe leads to a decrease of interplanar spacing, hence the decrease of crystal cell parameters. The full‐width at half‐maximum (FWHM) of the three strong peaks of FeFe‐PBA‐gel are 0.93°, 0.85°, and 0.72°, while that of the three strong peaks of NiFe‐PBA‐gel are 1.97°, 1.43°, and 1.28°. The significant increase in FWHM of NiFe‐PBA‐gel indicates a decrease in particle diameter. The Fourier transform infrared (FTIR) spectra of NiFe‐PBA‐gel and FeFe‐PBA‐gel indicate that they have similar functional groups (Figure 2b). The peaks of NiFe‐PBA‐gel at 3385, 2066, 1565, and 1378 cm^–1^ are assigned to O—H stretching mode, C≡N bending mode, C—H bending mode, and O—H bending mode.^[5,^ ^35^ ^]^

After annealing in argon and air, the XRD pattern of NiFe‐PBA‐gel‐cal (an Fe_3_O_4_/NiC*_x_* composite) is shown in Figure 2c, the diffraction peaks at 2*θ* = 32.6°, 36.8°, 44.7°, 59.4°, 64.9°, and 11.5° match well with the (220), (311), (400), (511), (440), and (533) crystal planes of Fe_3_O_4_ (JCPDS no: 26–1136), and the peaks at 2*θ* = 29.5°, 40.4°, 49.2° are assigned to the (111), (204), and (009) planes of NiC*_x_* (JCPDS no: 45–0979). While the calcined product of FeFe‐PBA‐gel (FeFe‐PBA‐gel‐cal) is single component Fe_3_O_4_. This suggests the Ni first combined with the C in the precursor under an argon atmosphere to form a strong Ni—C bond, which was not broken in the subsequent annealing in air, while Fe reacted with O to form Fe_3_O_4_, thereby forming the composite of NiC*_x_* and Fe_3_O_4_. In the FTIR spectrum of NiFe‐PBA‐gel‐cal, the peaks at 3073 and 548 cm^–1^ correspond to O—H stretching and Fe—O bending modes, and the additional peak at 1333 cm^–1^ is attributed to the Ni—C bond, indicating the formation of NiC*_x_* (Figure 2d). The nitrogen adsorption–desorption curves (Figure 2e) of NiFe‐PBA‐gel and NiFe‐PBA‐gel‐cal both demonstrate type IV isotherms, indicating the existence of mesopores.^[^ ^36^ ^]^ The specific surface areas of NiFe‐PBA‐gel and NiFe‐PBA‐gel‐cal calculated by the Brunauer–Emmett–Teller method are 122.7 and 431.3 m^2^ g^–1^, respectively. The pore volume was raised from 0.293 to 0.639 cm^3^ g^–1^ after the annealing. The pore sizes of the precursor were centered at 6.4 and 7.9 nm, while they shrink to 5.9 nm in NiFe‐PBA‐gel‐cal, suggesting the heating reduces the mesopore diameter (Figure S3, Supporting Information).

XPS was performed to analyze the composition of NiFe‐PBA‐gel and NiFe‐PBA‐gel‐cal. The survey spectra indicate that the annealed network is composed of C, O, Ni, and Fe elements only (Figure 2f), indicating the N of the original gel is oxidized under high‐temperature pyrolysis in air. From the fitted high‐resolution C 1s spectrum in Figure 2g, the peak at around 284.8 eV is assigned to C═C, two peaks of C—O and C—N disappear after the calcination, and an extra peak appeared at 281.1 eV corresponding to C—Ni, suggesting the formation of NiC*_x_*.^[^ ^37^, ^38^ ^]^ The N 1s spectrum (Figure 2h) of the PBA precursor was fitted with two peaks at 403.1 and 402.3 eV, corresponding to pyrrolic N and C≡N.^[^ ^39^ ^]^ The annealing in air caused loss of the pyrrolic N and C≡N peaks, consistent with the EDS result. The fitted Fe 2p XPS spectra are shown in Figure 2i, the coexistence of Fe^2+^ and Fe^3+^ appears in the calcined product, indicating the formation of Fe_3_O_4_, and movement of Fe^2+^ to low binding energy is due to the increase of the surface electron density caused by the acceptance of electrons from oxygen.^[^ ^40^ ^]^

### 2.2 Evaluation of Electrocatalytic OER Activity

**Figure 3:**
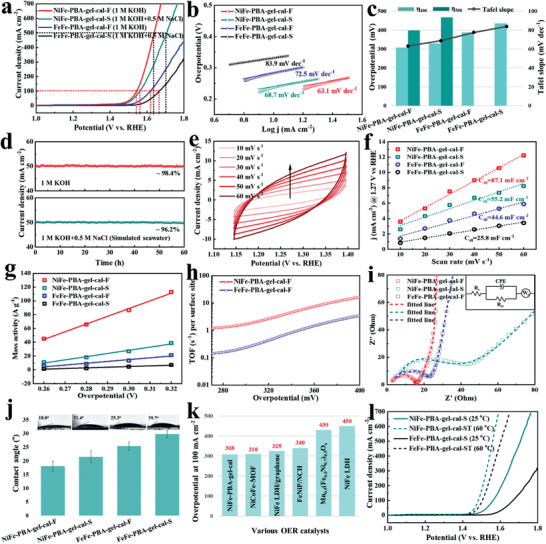
Oxygen evolution a) polarization curves and b) Tafel plots of NiFe‐PBA‐gel‐cal and FeFe‐PBA‐gel‐cal in both alkaline freshwater and alkaline simulated seawater. c) The Tafel slope and the overpotential comparison for NiFe‐PBA‐gel‐cal and FeFe‐PBA‐gel‐cal in electrochemical OER. d) Chronoamperometry stability test of NiFe‐PBA‐gel‐cal in both alkaline freshwater and alkaline simulated seawater at the potential of 1.52 and 1.53 V versus RHE, respectively. e) CV curves of NiFe‐PBA‐gel‐cal at different scan rates in alkaline simulated seawater. f) Capacitive current measured at 1.27 V of NiFe‐PBA‐gel‐cal and FeFe‐PBA‐gel‐cal as a function of scan rate. g) Mass activity of NiFe‐PBA‐gel‐cal and FeFe‐PBA‐gel‐cal in both alkaline freshwater and alkaline simulated seawater. h) TOF of NiFe‐PBA‐gel‐cal and FeFe‐PBA‐gel‐cal in both alkaline freshwater and alkaline simulated seawater. i) Nyquist plots of NiFe‐PBA‐gel‐cal and FeFe‐PBA‐gel‐cal in both alkaline freshwater and alkaline simulated seawater (inset: equivalent electric circuit for the EIS plots). j) Electrolyte contact angles of NiFe‐PBA‐gel‐cal and FeFe‐PBA‐gel‐cal. k) Comparison of OER catalytic activity with some recently reported NiFe‐based non‐noble metal catalysts in alkaline freshwater. l) Oxygen evolution polarization curves of NiFe‐PBA‐gel‐cal and FeFe‐PBA‐gel‐cal in alkaline simulated seawater in both room temperature (25 °C) and high temperature (60 °C).

The OER performances of the as‐prepared catalysts were measured in both alkaline freshwater (1 m KOH) and alkaline simulated seawater electrolytes (1 m KOH + 0.5 m NaCl) at room temperature (25 °C) using a three‐electrode configuration. The endnotes of “F” and “S” in the labels represent the measurements conducting in the freshwater and seawater, respectively. The linear sweep voltammetry (LSV) results are shown in **Figure** **3**a,c and Table S1 in the Supporting Information. NiFe‐PBA‐gel‐cal exhibited significantly improved OER activity compared to FeFe‐PBA‐gel‐cal in alkaline freshwater, NiFe‐PBA‐gel‐cal requires overpotentials as low as 308 and 398 mV to achieve current densities of 100 and 500 mA cm^–2^, smaller than that of FeFe‐PBA‐gel‐cal (387 mV at 100 mA cm^–2^). The OER activity of both the calcined PBA networks in alkaline seawater decreased, NiFe‐PBA‐gel‐cal and FeFe‐PBA‐gel‐cal required overpotentials of 329 (435) and 427 mV to drive current densities of 100 and 500 mA cm^–2^. The reduction of the catalytic activity in seawater is mainly due to the obstruction of active sites and surface poisoning by chlorides. The corresponding Tafel plots were calculated and are shown in Figure 3b,c and Table S1 in the Supporting Information, the Tafel slope values of NiFe‐PBA‐gel‐cal and FeFe‐PBA‐gel‐cal in alkaline freshwater and simulated seawater are 63.1, 77.5; 68.7, 83.9 mV dec^–1^, respectively. The relatively lower Tafel slopes of NiFe‐PBA‐gel‐cal indicate the generation of NiC*_x_* results in higher reaction activity and transfer coefficient.^[^ ^41^, ^42^ ^]^ Figure 3d shows the stability of NiFe‐PBA‐gel‐cal in freshwater and simulated seawater, as determined in a chronoamperometry test. Only a negligible decrease in current density was observed after 60 h measurement, maintaining ≈98.4% in freshwater and ≈96.2% in simulated seawater, showing the excellent durability of NiFe‐PBA‐gel‐cal. The produced gas was analyzed by time‐series mass spectrometry (MS), only O_2_ gas was detected, indicating no Cl_2_ gas was generated during the chronopotentiometry test (Figure S4, Supporting Information).

Cyclic voltammetry (CV) curves at different scan rates were performed for the evaluation of electrochemically active surface area (ECSA), which is proportional to the double‐layer capacitance (*C*_dl_). As shown in Figure 3e,f and Figure S5 in the Supporting Information, the C_dl_ of NiFe‐PBA‐gel‐cal in freshwater and seawater are 87.1 and 55.2 mF cm^–2^, exhibiting twice those of FeFe‐PBA‐gel‐cal, implying the high catalytic activity is created by the pseudo‐amorphous NiC*_x_*. To further assess the intrinsic catalytic activity, mass activity was calculated and compared for the annealed PBA products. The mass activity (Figure 3g) of NiFe‐PBA‐gel‐cal in freshwater is 66.3 A g^–1^ at an overpotential of 280 mV, which is approximately four times higher than that of NiFe‐PBA‐gel‐cal in simulated seawater, this may be due to the formation of hypochlorite attached to the surface of the network blocking combination with oxygen‐containing intermediates during the electrolysis.^[^ ^43^, ^44^, ^45^ ^]^ The mass activity of NiFe‐PBA‐gel‐cal is also over ten times that of FeFe‐PBA‐gel‐cal. Turnover frequencies (TOFs) for NiFe‐PBA‐gel‐cal and FeFe‐PBA‐gel‐cal were also calculated by using the ECSA at different overpotentials (Figure 3e,f and Figure S5, Supporting Information) based on the total deposited metal amount, a similar tendency as mass activity is observed for TOFs (Figure 3h). The TOFs of NiFe‐PBA‐gel‐cal are 1.33 s^–1^ at a low overpotential of 280 mV, which is 9.1 times greater than that of FeFe‐PBA‐gel‐cal. It is worth noting that the determined intrinsic activity values are inevitably underestimated because active sites are only endowed by the surface metal species, rather than the whole transition metal components.^[^ ^46^, ^47^, ^48^ ^]^ Electrochemical impedance spectroscopy (EIS) was measured and is shown in Figure 3i, where the reduced semicircle in the Nyquist plot of NiFe‐PBA‐gel‐cal compared with FeFe‐PBA‐gel‐cal indicates a decrease of interfacial charge transfer resistance (*R*_ct_) (Table S2, Supporting Information) and promotion of OER activity. Electrolyte contact angle measurements (Figure 3j) were performed to study wettability of the calcined products in both freshwater and simulated seawater. NiFe‐PBA‐gel‐cal displayed a higher hydrophilic property (contact angle, 18.0°) than that of FeFe‐PBA‐gel‐cal (contact angle, 25.3°) in freshwater. The remarkable OER activity of the as‐obtained electrocatalysts is superior to most of the state‐of‐the‐art NiFe‐based non‐noble metal OER electrocatalysts previously reported (Figure 3k and Tables S3 and S4, Supporting Information). Considering the outstanding catalytic performance of the calcined PBA catalysts for OER, they were applied under commercial electrolysis condition, which is usually with high temperature, and the continuous supply of seawater leads to the accumulation of salt, leading to high NaCl concentration. The label “ST” as an endnote represents measurements conducted in a simulated industry electrolysis condition (1 m KOH + 1 m NaCl, 60 °C). The results (Figure 3l) show that NiFe‐PBA‐gel‐cal exhibits better activity than FeFe‐PBA‐gel‐cal under the simulated industrial electrolysis environment, suggesting that Ni‐containing species hinder the corrosion by chlorides. It is also observed that FeFe‐PBA‐gel‐cal shows higher OER performance in the industrial environment compared to the room temperature, indicating that Fe_3_O_4_ activity is enhanced under high‐temperature conditions.

### 2.3 Active Sites for Oxygen Evolution Catalysis

#### 2.3.1 Post‐Mortem Investigations after OER

**Figure 4:**
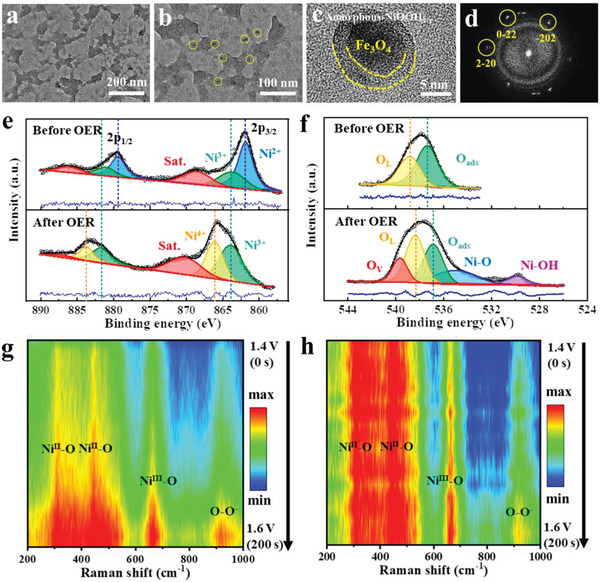
Active sites identification for OER. a–c) TEM images of NiFe‐PBA‐gel‐cal after OER test. d) SAED pattern of (c). XPS high‐resolution spectra at e) Ni 2p edge and f) O 1s edge of NiFe‐PBA‐gel‐cal before and after OER test. The spectra were shown with raw data and fitting data derived by Lorentz–Gaussian function. g) Operando Raman spectra contour plot of NiFe‐PBA‐gel‐cal obtained from the voltage increasing from 1.4 to 1.6 V (vs RHE) and h) the corresponding contour plot normalized by the value at 446 cm^–1^.

To gain an understanding of the catalytic active sites that give rise to extraordinary OER activity of the NiFe‐PBA‐gel‐cal catalyst, the nanostructure, surface composition, and chemical state evolution after the OER test were studied. The TEM images of NiFe‐PBA‐gel‐cal after OER are shown in **Figure** **4**a,b, the original network structure is thinner. From the HRTEM image (Figure 4c), it is worth noting that Fe_3_O_4_ nanocrystals have become core–shell composite structures, where the core is crystalline Fe_3_O_4_, and the shell is amorphous material. The corresponding selected area electron diffraction (SAED) pattern (Figure 4d) is composed of single‐crystal lattices and amorphous rings. The amorphous shell is further verified by XPS, STEM‐mapping, and operando Raman spectroscopy.

By comparing the Ni 2p XPS spectra (Figure 4e) before and after the OER test, it is found that Ni^2+^ and Ni^3+^ in the metastable pseudo‐amorphous NiC*_x_* were oxidized to Ni^3+^ and Ni^4+^.^[^ ^49^ ^]^ The shift toward low binding energy of the two peaks (lattice oxygen and adsorbed oxygen) in the fitted O 1s spectra (Figure 4f) also proves the increased valence states of nickel, suggesting the surface electron density of NiFe‐PBA‐gel‐cal increases during OER, which enhances the adsorption of hydroxyl species and promotes catalytic activity.^[^ ^50^ ^]^ Moreover, the appearance of the peaks attributed to Ni—O and Ni—OH suggests the formation of nickel (oxy)hydroxides.^[^ ^51^, ^52^ ^]^ Combined with the TEM result, it is deduced that the amorphous shell stems from in situ formed nickel (oxy)hydroxides. The emergence of the oxygen vacancy (Ov) peak is due to the formation of in situ transformation of metastable pseudo‐amorphous NiC*_x_* to nickel (oxy)hydroxides, which causes the escape of oxygen from the crystal lattice and leads to the generation of a large number of defects and oxygen deficiency, serving as active sites for OER.^[^ ^53^ ^]^ From the STEM‐mapping of NiFe‐PBA‐gel‐cal after water oxidation (Figure S6, Supporting Information), it is observed that the ratios of Ni, Fe, and O elements increase, while that of C significantly decreases compared with the catalyst before OER, further proving the conversion from NiC*_x_* to nickel (oxy)hydroxides.

Operando Raman spectroscopy (Figure 4g) was conducted to elucidate the real‐time evolution of Ni ions during the OER process. The intensity of the spectra of NiFe‐PBA‐gel‐cal gradually increases as the voltage increases from 1.4 to 1.6 V, this could be due to the surface reconstruction induced by high voltage. The peaks at 446 and 661 cm^–1^ are attributed to the A_1g_ stretching vibrations of Ni—O in Ni(OH)_2_ and NiOOH, respectively.^[^ ^54^ ^]^ In order to reduce the influence of the rise of the base of the spectra on the peaks, the data were normalized by the value at 446 cm^–1^, the contour plot is shown in Figure 4h. The decrease in the relative intensities (*I*_446_/*I*_661_) indicates an increase of NiOOH with the rise of voltage.^[^ ^55^ ^]^ The appearance of the peak of O—O^–^ with the increase of potential also suggests the participation of lattice oxygen.^[^ ^53^, ^54^, ^55^ ^]^ It is worth noting that as the voltage decreases, the peak intensity gradually returns to the original, suggesting that the surface reconstruction is reversible (Figure S7, Supporting Information).

Combining the results above after OER test, it is concluded that the amorphous shell is NiOOH_2−_ *_x_* evolved from the pseudo‐amorphous NiC*_x_* network during OER electrocatalysis. The formation of high‐valent nickel states serves as active sites and facilitates electron transfer from the shell to core and catalyzes the water oxidation. The in situ generated amorphous NiOOH_2−_ *_x_* shells play a role as a buffer layer to reduce the contact between chloride ions and Fe_3_O_4_ and protect the overall activity of the catalyst, contributing to the superior durability during seawater electrolysis.

Furthermore, the role of Fe_3_O_4_ in OER has also been studied, it is observed from the XRD pattern (Figure S8, Supporting Information) of NiFe‐PBA‐gel‐cal after OER that the peaks of Fe_3_O_4_ remain unchanged, while the peaks of NiC*_x_* disappear. The significant reduction of carbon content in the EDS‐mapping after OER also reflects the conversion of NiC*_x_* (Figure S6, Supporting Information). In addition, from the Fe 2p XPS spectrum of NiFe‐PBA‐gel‐cal (Figure S9, Supporting Information), the peaks of Fe 2p are composed of Fe^3+^ and Fe^4+^, which remain unchanged after OER, indicating that crystalline Fe_3_O_4_ in the catalyst is relatively stable during water oxidation in the existence of pseudo‐amorphous NiC*_x_*, and is not prone to self‐reconstruct for the conversion to iron (oxy)hydroxide. Therefore, the active species of NiFe‐PBA‐gel‐cal for the higher water oxidation activity compared with FeFe‐PBA‐gel‐cal is the nickel (oxy)hydroxide transformed from NiC*_x_*.

#### 2.3.2 Interpretation of the Role of O 2p

**Figure 5:**
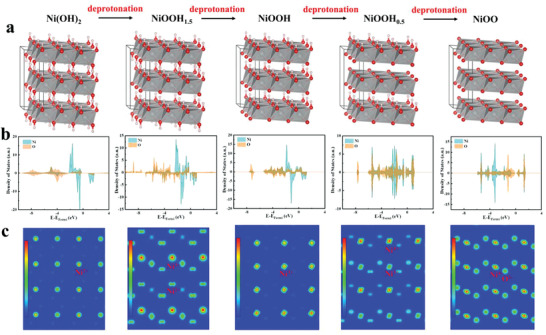
Geometric and electronic structure evolution during OER. a) Geometric structure, b) electronic structure, and c) charge density evolution in the multistep deprotonation process from Ni(OH)_2_ to NiOO during OER.

To shed more light on the catalytic nature, DFT calculations were used to track the evolution of the catalyst's electronic structure during the surface reconstruction (**Figure** **5**a and Figure S10, Supporting Information). The valence band maximum (VBM) and the conduction band minimum (CBM) of the five nickel compounds with deprotonation at Gamma point are presented in the projected density of states (PDOS; Figure 5b). It is observed that Ni(OH)_2_ is an insulator with a band gap of ≈2.35 eV. The deprotonation process requires Ni to denote one electron to the more electronegative oxygen atom, causing the charge state of Ni to increase from +2 to +3, and the fully deprotonated NiOO has Ni of +4 charge state. With deprotonation, the insulating nature of Ni(OH)_2_ diminishes, the O 2p component near the Fermi level increases sharply, and the O 2p band center gradually shifts to higher energy, indicating increased conductivity. The distribution of charge densities on the Ni metal center and the surrounding ligands is demonstrated in the partial charge density diagram (Figure 5c), it is observed that the contribution of oxygen gradually increases and electrons are drawn more toward the oxygen ligands with deprotonation. In the full deprotonated system, it is clear to identify the strong *π* accepting nature of O^2–^ ligand group.

**Figure 6:**
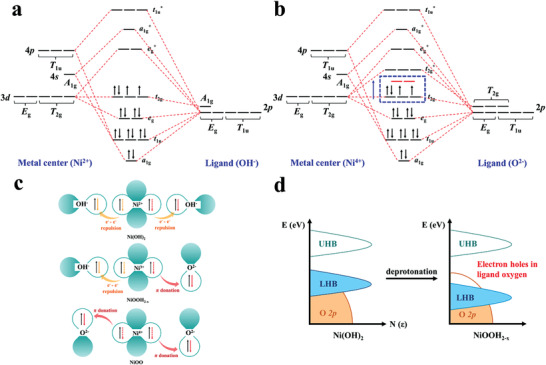
Formation of electron holes in O 2p. a) Molecular orbital diagram of Ni(OH)_2_ in the influence of 6 OH^–^ ligands. b) Molecular orbital diagram of NiOO in the influence of 6 O^2–^ ligands. Orbitals in red indicated the nonbonding state orbitals when the deprotonation process is incomplete, the nonbonding state orbitals act as VBM in the system. c) The electron coupling schematic representations of Ni compounds during deprotonation. d) Schematic band diagrams for Ni compounds upon deprotonation.

The detailed molecular orbital (MO) diagram and electron coupling for Ni(OH)_2_ are shown in **Figure** **6**a,c. Ni(OH)_2_ forms an octahedral structure with six hydroxyl groups, which act as ligands and surround the core Ni metal center. The overall structure of Ni(OH)_2_ processes *O_h_* group geometry, where Ni 4*s* orbital has symmetry *A*_1g_, Ni 3d orbitals have symmetry *T*_2g_ and *E*_g_, and Ni 4p orbital has symmetry *T*_1u_. With all six hydroxyl group ligands, there is no matching orbital in the ligand side to couple with Ni 3d *T*_2g_ orbitals. This leaves three Ni 3d *T*_2g_ orbitals hanging in the MO diagram, and these nonbonding orbitals turn into VBM. CBM is composed of the antibonding *e*_g_* orbitals resulting from couplings between Ni 3d orbitals and *σ* orbitals of ligands, and this leads to a large charge transfer energy between VBM and CBM.

The MO diagram and electron interactions of fully deprotonated structure NiOO are shown in Figure 6b,c. With full hydrogen vacancies, the 3d orbitals of Ni could fully couple with O^2–^ 2p *π* orbitals. The VBM and CBM turn into the bonding state and antibonding state of *t*_2g_ molecule orbitals, respectively. The charge transfer energy turns out to be smaller than that in the six hydroxyl ligands, and the atomic orbitals of oxygen shifted up compared with that of OH^–^, which can be visualized in the PDOS calculation in Figure 5b. For the partially deprotonated Ni(OH)_2_, the ligands are composed of OH^–^ group and O^2–^. OH^–^ group is a strong *σ* donor and O^2–^ is a strong *π* acceptor, and only O^2–^ orbitals contain *T*_2g_ symmetry that could couple with Ni 3d orbitals. With a deficient number of O^2–^ *π* orbitals, some of the *3*d orbitals of Ni are left as nonbonding orbitals. The energy level of the nonbonding orbitals is the same as the atomic orbitals from Ni 3d, and thus it lies in between the bonding and antibonding orbitals of *t*_2g_ and *t*_2g_*. The nonbonding orbitals become the VBM of the system, and charge transfer energy between VBM and CBM in the system dramatically decreases. It is known from Mott–Hubbard theory that the antibonding orbitals will be split into one empty upper‐Hubbard band (UHB) one filled lower‐Hubbard band (LHB) under d‐d orbital Coulomb interaction (*U*).^[^ ^56^ ^]^ When the charge transfer energy is much smaller than that of *U*, LHB states lose electrons and the energy level shifts downward, and this leads to the formation of electron holes on the O 2p orbitals.

The schematic band diagrams of the underlying mechanism are displayed in Figure 6d. The presence of localized O 2p electron holes implies that the oxidized oxygen ions have an unpaired electron. High‐valence states of Ni play a role in activating these oxidized oxygen ions, which act as electrophilic centers for OER.

### 2.4 In‐Depth Understanding of OER Pathway

**Figure 7:**
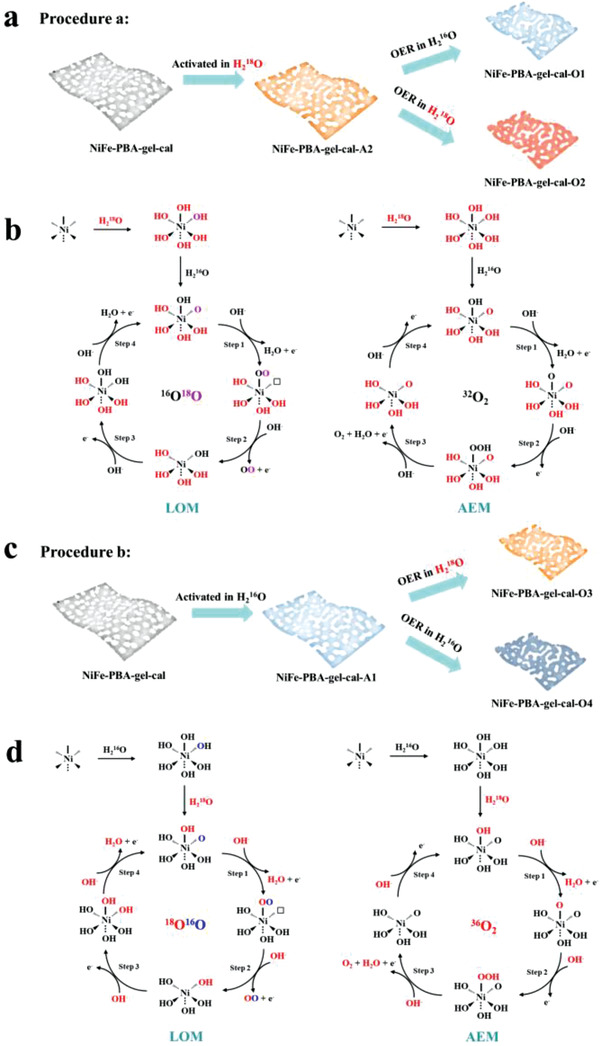
OER mechanisms with concerted and nonconcerted proton–electron transfer. a) Schematic illustration of the isotopic labeling experiments of procedure a, where H_2_^18^O was used to activate the catalyst and H_2_^16^O served as electrolyte for the oxygen evolution. b) The corresponding possible nonconcerted proton–electron transfer OER mechanism that evolves ^16^O^18^O and proton–electron transfer OER mechanism that evolves ^32^O_2_ (^16^O^16^O) (the black, purple, and red colors mark the chemically inert lattice oxygen, active lattice oxygen involving OER, and oxygen from the electrolyte, respectively. □ represents lattice O_v_). c) Schematic illustration of the isotopic labeling experiments of procedure b, where H_2_^16^O was used to activate the catalyst and H_2_^18^O served as electrolyte for the oxygen evolution. d) The corresponding possible nonconcerted proton–electron transfer OER mechanism that evolves ^18^O^16^O and proton–electron transfer OER mechanism that evolves ^36^O_2_ (^18^O^18^O).

Due to the generation of oxidized nickel species with high valence state and the in situ formed amorphous shells including rich oxygen defects, lattice oxygen is easily activated and participates in the OER process, greatly promoting its activity.^[^ ^57^ ^]^ To determine the reaction mechanism in the high‐performance catalysts, two complementary isotopic procedures a and b, were conducted and are illustrated in **Figure** **7**a,c. In procedure a, ^18^O‐based ultrapure water was first used to activate NiFe‐PBA‐gel‐cal to generate Ni(OH)_2_ through cycling, and then the activated material was employed in OER in an ^16^O‐based electrolyte, the obtained material was denoted as NiFe‐PBA‐gel‐cal‐O1, and the catalysis performed in H_2_^18^O was used as a comparison, named NiFe‐PBA‐gel‐cal‐O2. Procedure b is opposite, ^16^O was incorporated into NiFe‐PBA‐gel‐cal, and the activated material was employed in OER in an ^18^O‐based electrolyte and named NiFe‐PBA‐gel‐cal‐O3, the control sample activated and oxidized in an ^16^O‐based electrolyte was denoted NiFe‐PBA‐gel‐cal‐O4.

**Figure 8:**
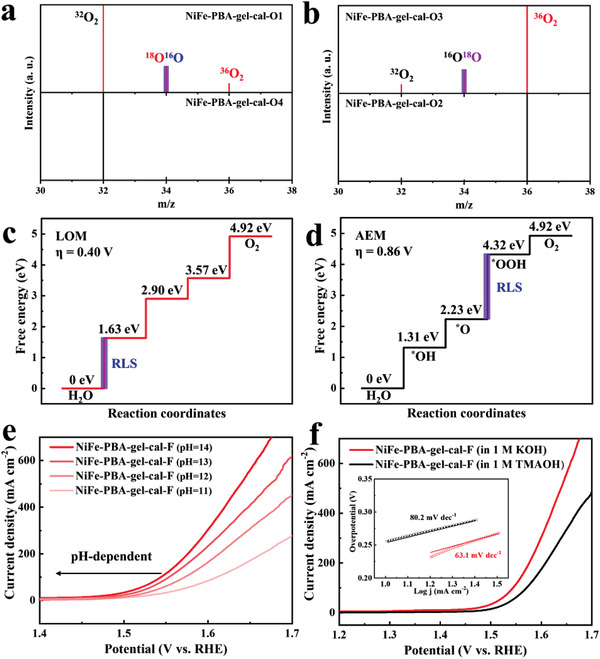
Investigation of the proposed OER pathway. a) The detected MS signals of the generated oxygen molecules with isotope‐labeling experiments of NiFe‐PBA‐gel‐cal‐O1 and NiFe‐PBA‐gel‐cal‐O4, MS signals were normalized by initial intensity of ^32^O_2_. b) The detected MS signals of the generated oxygen molecules with isotope‐labeling experiments of NiFe‐PBA‐gel‐cal‐O3 and NiFe‐PBA‐gel‐cal‐O2, MS signals were normalized by initial intensity of ^36^O_2_. c) Gibbs free energy diagram of LOM pathway of NiFe‐PBA‐gel‐cal after OER test. d) Gibbs free energy diagram of AEM pathway of NiFe‐PBA‐gel‐cal after OER test. e) LSV curves of NiFe‐PBA‐gel‐cal showing variation of OER performance with respect to pH. f) LSV curves of NiFe‐PBA‐gel‐cal in 1 m KOH (red) and 1 m TMAOH (black). Inset shows the corresponding Tafel plots.

The generated gas was tested by operando mass spectrometry (**Figure** **8**a,b), the significant increase in the intensity of the ^16^O^18^O and ^36^O_2_ signals in NiFe‐PBA‐gel‐cal‐O1 compared with those of natural abundance in NiFe‐PBA‐gel‐cal‐O4, and the similar increase of the ^18^O^16^O and ^32^O_2_ signals in NiFe‐PBA‐gel‐cal‐O3 in comparison with those of natural abundance in NiFe‐PBA‐gel‐cal‐O2 unambiguously corroborates the participation of lattice oxygen ligands in oxygen evolution at NiFe‐PBA‐gel‐cal.

Figure 7b,d is the proposed reaction mechanisms involving LOM and adsorbate evolution mechanism (AEM) to explain the detection of ^34^O_2_ in the two isotope‐labeling experimental procedures. The Ni(OH)_2_ is oxidized to NiOOH to participate in the reaction via surface self‐reconstruction. The AEM pathway for NiOOH includes four elementary steps and three different intermediates, *OH, *O, and *OOH. In LOM, the formation of ^34^O_2_ can be explained by charge transfer steps on surface oxygen sites. Different from AEM, LOM takes the single metal site as the catalytic center to adsorb OH and follows the deprotonation step. Step II is a chemical step to produce molecular ^34^O_2_. The surface reconstruction enables the direct coupling of the *O intermediate and activated lattice oxygen to form ^16^O—^18^O, which is energetically conducive to the high‐valence Ni cations. The ^16^O—^18^O is a newly formed O_2_ molecule to undergo the subsequent oxygen evolution. The generation of ^36^O_2_ in NiFe‐PBA‐gel‐cal‐O1 is due to the formation of an ^18^O—^18^O bond from two surface oxygen ions of the oxide in the ^18^O‐based electrolyte. The similar production of ^32^O_2_ in NiFe‐PBA‐gel‐cal‐O3 in the H_2_^16^O electrolyte is also because ^16^O—^16^O is formed, and the two procedures complement each other, which strongly prove LOM for NiFe‐PBA‐gel‐cal.

Furthermore, the Gibbs free energies of the active species NiOOH in each step for different pathways were calculated and are shown in Figure 8c,d, the results indicate that it is more thermodynamically favorable to perform OER for NiFe‐PBA‐gel‐cal through LOM pathway, the overpotential of the rate‐limiting step (RLS) for NiFe‐PBA‐gel‐cal through LOM is only 0.40 V, lower than that through AEM (0.86 V).

To further verify the LOM for NiFe‐PBA‐gel‐cal, OER performance was also tested on the dependence of pH. The OER performance of LOM is highly correlated with pH because its RLS is the deprotonation of hydroxyl groups, and the p*K*_a_ of the surface deprotonation eventually leads to a pH‐dependence.^[^ ^58^, ^59^ ^]^ While the conventional AEM is composed of four concerted proton–electron transfer steps on surface metal centers, yielding pH‐independent activity at the RHE scale.^[^ ^60^, ^61^ ^]^ As presented in Figure 8e, the onset potential of NiFe‐PBA‐gel‐cal decreases with increasing pH, indicating the underlying LOM pathway. Moreover, distinguished from AEM, peroxo‐like (O_2_^2–^) and superoxo‐like (O_2_^–^) species are produced on the surface of catalyst in LOM, which specifically interact with tetramethylammonium cation (TMA^+^), and thus tetramethylammonium hydroxide (TMAOH) was used to replace KOH as the electrolyte to confirm the reaction mechanism. It was observed the OER activity of NiFe‐PBA‐gel‐cal significantly reduced in 1 m TMAOH compared with that in 1 m KOH, and the Tafel slope increased from 63.1 to 80.2 mV dec^–1^ (Figure 8f), suggesting that the strong binding of TMA^+^ with the negatively charged oxygenated species restrained the OER via the LOM. While FeFe‐PBA‐gel‐cal displays little OER performance change in 1 m TMAOH and 1 m KOH (Figure S11, Supporting Information), indicating the composite of the pseudo‐amorphous NiC*_x_* in NiFe‐PBA‐gel‐cal facilitates LOM, greatly alters the electron transfer path. In addition, operando Raman spectroscopy was also preformed to verify the participation of lattice oxygen species (Figure S12, Supporting Information). The H_2_^16^O‐activated NiFe‐PBA‐gel‐cal was subjected to potentiation electrolysis at 1.6 V versus reversible hydrogen electrode (RHE). After electrolysis in H_2_^18^O, the peak at 446 cm^–1^ related to the A_1g_ vibrational mode of Ni^II^‐O shifted by ≈13 cm^–1^ compared to the peak from ^16^O‐labeled sample, while the peak of O—O^–^ shifted by ≈25 cm^–1^, indicating the replacement of two ^16^O atoms by two ^18^O atoms, which demonstrates the participation of lattice oxygen and potential LOM pathway.

### 2.5 HER Electrolysis and Overall Seawater Splitting

#### 2.5.1 Evaluation of HER and Seawater Splitting Performance

**Figure 9:**
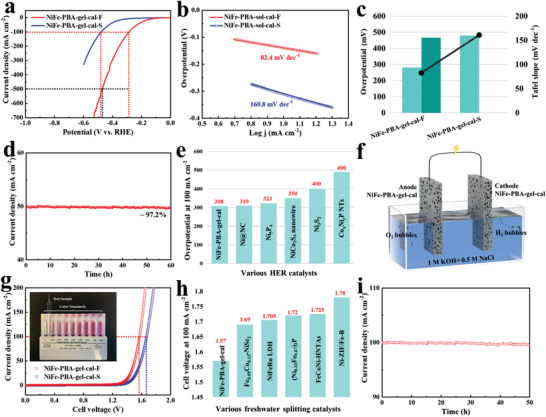
Electrocatalytic HER and water‐splitting measurements in freshwater and simulated seawater. Hydrogen evolution a) polarization curves and b) Tafel plots of NiFe‐PBA‐gel‐cal in alkaline freshwater and simulated seawater. c) The Tafel slope and the overpotential comparison for NiFe‐PBA‐gel‐cal in alkaline freshwater and simulated seawater. d) Chronoamperometry stability test of NiFe‐PBA‐gel‐cal in alkaline freshwater at the potential of −0.24 V versus RHE. e) Comparison of HER catalytic activity with some recently reported Ni‐based non‐noble metal catalysts in alkaline freshwater. f) Schematic illustration of the water‐splitting electrolyzer using NiFe‐PBA‐gel‐cal for both electrodes. g) Overall water‐splitting performance of NiFe‐PBA‐gel‐cal//NiFe‐PBA‐gel‐cal electrode couple in alkaline freshwater (red) and simulated seawater (blue) (inset: hypochlorite detection result of the electrolyte after seawater splitting stability test). h) Comparison of overall water‐splitting activity with some recently reported NiFe‐based non‐noble metal catalysts in alkaline freshwater. i) Chronoamperometry stability test for NiFe‐PBA‐gel‐cal//NiFe‐PBA‐gel‐cal electrode couple at a voltage of 1.57 V.

The HER performance of NiFe‐PBA‐gel‐cal in alkaline freshwater and simulated seawater was also measured with LSV and labeled with “F” and “S,” respectively. As shown in **Figure** **9**a,c and Table S5 in the Supporting Information. NiFe‐PBA‐gel‐cal exhibits remarkable HER catalytic activity in alkaline freshwater, with overpotentials of 281 and 466 mV reaching current densities of 100 and 500 mA cm^–2^. Tafel slope values (Figure 9b,c and Table S5, Supporting Information) of NiFe‐PBA‐gel‐cal in alkaline freshwater and simulated seawater are 82.4 and 160.8 mV dec^–1^, respectively. The value in the range of 40–120 mV dec^–1^ suggests the reaction on the surface follows the Volmer–Heyrovsky mechanism, while that above 120 mV dec^–1^ is attributed to Volmer–Tafel mechanism.^[^ ^50^ ^]^ In alkaline freshwater, one adsorbed M‐H* on the surface binds to H_2_O and then accepts another e^–^ to form OH^–^ and H_2_, while in alkaline seawater, two adsorbed M‐H* directly combine for the formation of H_2_. This indicates the addition of chloride changes the HER pathway, resulting in high adsorption free energy and weak bonding with M‐H*, which reduces the HER activity. Moreover, NiFe‐PBA‐gel‐cal shows good stability at a current density of 50 mA cm^–2^ in alkaline freshwater, retaining 97.2% over a 60 h test (Figure 9d). The high HER activity of NiFe‐PBA‐gel‐cal is comparable to most of the previously reported Ni‐based non‐noble metal catalysts (Figure 9e and Table S6, Supporting Information). Considering the outstanding catalytic performance of NiFe‐PBA‐gel‐cal for both OER and HER, the overall freshwater and alkaline seawater splitting performance was further investigated by integrating the catalysts into a two‐electrode alkaline electrolyzer, in which NiFe‐PBA‐gel‐cal was used as both the cathode and anode (Figure 9f). As displayed in Figure 9g, the cell voltages needed to produce a current density of 100 mA cm^–2^ are as low as 1.57 and 1.66 V in alkaline freshwater and simulated seawater electrolysis, respectively, showing excellent water‐splitting activity, which outperforms that of most NiFe‐based non‐noble metal catalysts (Figure 9h and Table S7, Supporting Information). The electrolyzer retains outstanding durability with no noticeable degradation over 50 h chronoamperometry operation at a cell voltage of 1.57 V in alkaline freshwater electrolytes (Figure 9i), and the cell voltage shows only slight increase after 30 h chronopotentiometry stability test at a higher current density of 500 mA cm^–2^ (Figure S14, Supporting Information).

The possible formation of Cl^–^ oxidation product (ClO^–^) during the seawater splitting was examined using the *N*,*N*‐diethyl‐*p*‐phenylenediamine (DPD) method, DPD reacts with hypochlorite ions and/or hypochlorous acid to acquire a pink color, which is quantitatively consistent with the concentration of these substances in solution within the working range, the absence of pink indicates that no hypochlorite ions or hypochlorous acid is formed in the reaction solution (Figure 9g, inset).

The performance of water splitting in alkaline real seawater (1 m KOH + real seawater) has been tested, and the results (Figure S15, Supporting Information) show that the cell voltage at the current density of 100 cm^–2^ is 1.64 V, suggesting a better activity than that in alkaline simulated seawater (1 m KOH + 0.5 m NaCl). The stability tests in both alkaline real seawater and simulated seawater have also been conducted and compared in Figure S16 in the Supporting Information, it is found that NiFe‐PBA‐gel‐cal shows slight attenuation after 30 h test in both real seawater and simulated seawater, indicating good durability of the catalyst. While the stability in real seawater is slightly poorer than that in simulated seawater, this is because the impurities, various metallic, and nonmetallic elements in real seawater deteriorate the corrosion of the catalyst, leading to its partial poisoning and deactivation.

#### 2.5.2 Active Sites for Hydrogen Evolution Catalysis

**Figure 10:**
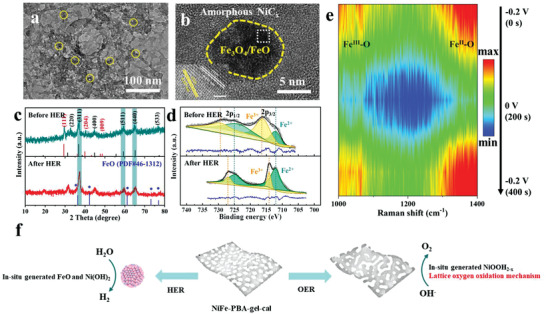
Active sites identification for HER and schematic representation of water‐splitting mechanism. a,b) TEM images of NiFe‐PBA‐gel‐cal after HER test (inset: HRTEM image of the white‐dotted square area in (b). c) XRD patterns of NiFe‐PBA‐gel and NiFe‐PBA‐gel‐after HER test. d) XPS high‐resolution spectra at Fe 2p edge of NiFe‐PBA‐gel‐cal and NiFe‐PBA‐gel‐after HER test. The spectra were shown with raw data and fitting data derived by Lorentz–Gaussian function. e) Operando Raman spectra contour plot of NiFe‐PBA‐gel‐cal obtained from the voltage increasing from 0 to −0.2 to 0 V (vs RHE). f) Schematic diagram of overall water‐splitting mechanism of the bifunctional NiFe‐PBA‐gel‐cal catalyst.

To disclose the active sites for HER, the post‐mortem investigations were performed on NiFe‐PBA‐gel‐cal after HER. TEM and HRTEM images (**Figure** **10**a,b) reveal the generation of FeO, the small peaks emerging at 36.4°, 42.3°, 61.2°, 73.1°, and 76.9° in the XRD pattern (Figure 10c) after HER correspond to the (111), (200), (220), (311), and (222) crystalline planes of FeO (JCPDS card no. 46–1312). From the Fe 2p high‐resolution XPS spectra in Figure 10d, it is observed that the proportion of Fe^2+^ increases while Fe^3+^ decreases after the HER test, indicating that part of the Fe^3+^ is reduced. The Fe^3+^ peaks also shift to low binding energy, indicating a decrease in surface electron density during HER, which reduces the adsorption free energy with H* and promotes its HER activity. Operando Raman spectra (Figure 10e and Figure S17, Supporting Information) reveal the increase of the relative intensity (*I*_FeII‐O_/*I*_FeIII‐O_) of Fe^II^‐O and Fe^III^‐O with the increase in the absolute value of the applied voltage, indicating the increase of the relative content of Fe^II^‐O in comparison to Fe^III^‐O. When the applied voltage returns to the initial value, the intensities of Fe^II^‐O and Fe^III^‐O remain similar as the original, indicating that the transformation of Fe^2+^ and Fe^3+^ is reversible. Moreover, the role of NiC*_x_* in HER has also been investigated. XPS spectra at Ni 2p and O 1s edge of the catalyst after HER were conducted (Figure S18, Supporting Information). It is observed that the Ni 2p peak in the XPS spectrum is basically the same as the one before HER, consisting of Ni^2+^ and Ni^3+^ ions, while the peak of Ni—O appears in the O 1s spectrum after HER, indicating the formation of nickel hydroxide, this could be because the applied potential in the alkaline media promotes the conversion of NiC*_x_* to Ni(OH)_2_. The HER performance of FeFe‐PBA‐gel‐cal has been conducted (Figure S19, Supporting Information), which shows a poorer activity than NiFe‐PBA‐gel‐cal, and thus is concluded that the improved electrocatalytic HER performance of NiFe‐PBA‐gel‐cal stems from the generated FeO and Ni(OH)_2_. According to the results above, the schematic diagram of the overall water‐splitting mechanism of the bifunctional NiFe‐PBA‐gel‐cal catalyst is shown in Figure 10f.

## 3 Conclusions

In summary, the ultra‐large 2D PBA network was synthesized by the sol–gel method, and then continuously calcined in different atmospheres for the preparation of the NiFe‐PBA‐gel‐cal catalyst with excellent seawater splitting performance. Detailed post‐mortem characterizations showed that after the OER test, the Fe_3_O_4_ particles originally dispersed on the NiC*_x_* network formed a core–shell structure. Operando Raman spectroscopy and XPS revealed that the core–shell structure is the in situ generated Fe_3_O_4_@NiOOH_2−_ *_x_* converted from the surface reconstruction of NiC*_x_*, which contains high‐valence ions and a large number of oxygen defects. DFT calculations and LFT reveal that the generated high valence states of Ni trigger the generation of local O *2*p holes, which acts as electrophilic centers for activating the OER redox reaction, greatly improving its electrochemical activity. Meanwhile, in situ isotope labeling of ^18^O and the replacement of KOH with TMAOH as an electrolyte reveal that the OER performance of NiFe‐PBA‐gel‐cal is intrinsically dominated by LOM pathway due to high‐valence nickel cations and abundant oxygen defects, which bypasses the adsorption of oxygen‐containing intermediates and facilitates the reaction kinetics. The catalyst after HER was also characterized in detail, and the results reveal the reduction from Fe^3+^ to Fe^2+^ in Fe_3_O_4_ and the evolution from NiC*_x_* to Ni(OH)_2_ leads to high catalytic performance.

## Conflict of Interest

The authors declare no conflict of interest.

## Supporting information

Supporting Information

## Data Availability

Research data are not shared.
