# Supplementary material for: A Self‐Reconstructed Bifunctional Electrocatalyst of Pseudo‐Amorphous Nickel Carbide @ Iron Oxide Network for Seawater Splitting
Source: Adv Sci (Weinh). 2022 Mar 25;9(15):2200146. doi: 10.1002/advs.202200146 (PMC9131433; doi:10.1002/advs.202200146)
Supplement: Supplementary file 1 — Supporting Information [file ADVS-9-2200146-s001.pdf]

ADVANCED SCIENCE
Open Access

Supporting Information

for *Adv. Sci.*, DOI 10.1002/advs.202200146

A Self-Reconstructed Bifunctional Electrocatalyst of Pseudo-Amorphous Nickel Carbide @ Iron Oxide Network for Seawater Splitting

*Hao Zhang, Songyuan Geng, Mengzheng Ouyang, Hossein Yadegari, Fang Xie and D. Jason Riley**

Supporting Information

A Self-Reconstructed Bifunctional Electrocatalyst of Pseudo-Amorphous Nickel Carbide @ Iron Oxide Network for Seawater Splitting

Hao Zhang[1], Songyuan Geng[2], Mengzheng Ouyang[3], Hossein Yadegari[1], Fang Xie[1], D. Jason Riley[1,*]

[1]Department of Materials and London Center for Nanotechnology, Imperial College London, London SW7 2AZ, U.K. E-mail: jason.riley@imperial.ac.uk

[2]Department of Chemistry, Imperial College London, London, SW7 2AZ UK.

[3]Department of Earth Science and Engineering, Imperial College London, London, SW7 2AZ UK.

Experimental section

Materials and methods

Materials: The following reagents were obtained and used as received without further processing: nickel(II) sulfate hexahydrate (≥ 98.5%), iron(II) sulfate heptahydrate (> 98.5%), sodium citrate dihydrate (≥ 99.0%) and potassium hexacyanoferrate(II) (> 99.0 %) were bought from Sigma-Aldrich. Absolute ethanol was purchased from VWR. Reverse Osmosis water (> 18.2 MOhms cm^{-1}) was utilized in all experimental procedures.

Preparation of NiFe-PBA-gel: Solution A was prepared by dissolving 0.6 mmol nickel(II) sulfate hexahydrate and 1.2 mmol sodium citrate dihydrate in 25 mL deionized water. Solution B was prepared by dissolving 0.4 mmol of potassium hexacyanoferrate(II) in 25 mL of deionized water. Then, solution B was added to solution A dropwise under magnetic stirring for 10 min, and the resultant mixed solution was aged for 24 h at room temperature. The gel was collected by centrifugation, washed several times with ethanol, and frozen-dry overnight to obtain a xerogel.

Preparation of FeFe-PBA-gel: The same procedures as for the preparation of NiFe-PBA-gel were followed, except that nickel(II) sulfate hexahydrate was replaced with iron(II) sulfate heptahydrate.

Preparation of NiFe-PBA-gel-cal: The NiFe-PBA-gel xerogel was collected and calcined in argon at 350 °C for 3 h at a heating rate of 2 ^{o}C min^{-1}, and then the argon was turned off and the calcined product was further annealed in air at 600 ^{o}C for 3 h with a ramping rate of 5 ^{o}C min^{-1}. The obtained black powders were collected after cooling to room temperature, washed several times with ethanol, and vacuum dried at 40 ^{o}C overnight.

Preparation of FeFe-PBA-gel-cal: The same procedures as for the preparation of NiFe-PBA-gel-cal were followed, except that NiFe-PBA-gel was replaced with FeFe-PBA-gel.

Characterizations

Powder X-ray diffraction (XRD) patterns were collected on a Bruker D2 ADVANCE diffractometer with Cu K_{α} radiation (λ=1.5418Å). The structure and morphology of the samples were characterized by field-emission scanning electron microscopy (FESEM, Zeiss LEO 1525) and transmission electron microscopy (TEM, JEOL-2100Plus). Energy-dispersive X-ray spectroscopy (EDS) attached to the TEM was used to analyze the composition of the nanoscale samples. The N_2 adsorption-desorption isotherm was measured at 77 K using a Quantachrome

Instruments Autosorb AS-6B. The samples were degassed in N_2 at 80 ^oC for 8 h before the measurements. The specific surface area was determined by the multi-point Brunauer-Emmett-Teller (BET) method and the pore-size distribution was calculated based on the Barrett-Joyner-Halenda (BJH) method. XPS analysis was conducted on a PHI-5000 VersaProbe X-ray photoelectron spectrometer using an Al Kα X-ray source. *Operando* Raman spectra was obtained with an inVia Renishaw confocal Raman microscope operated with an incident laser beam at 532 nm focused through a 50x objective (Leica). The laser intensity was set to < 1 mW and Raman spectra were collected in static mode, with an exposure time of few seconds every 5 minutes to minimize the sample heating. To monitor the evolution of catalyst samples during OER process, each Raman spectrum was collected after a constant potential was applied to the catalyst electrode for 5 min. Each Raman spectrum was obtained using an integration time of few seconds with accumulating 5 times. The laser shutter remained closed between spectrum collections. The gas product was extracted and analyzed using gas chromatography-mass spectrometry (GC-MS, 7890A and 5975C, Agilent). Static contact angles were measured with a contact angle meter, OCA20.

Electrochemical measurements

The electrochemical tests of the catalysts were performed using a Metrohm Autolab electrochemical workstation PGStat-12 (Utrecht, the Netherlands) connected to a three-electrode cell. A glassy carbon electrode (GCE) of 3 mm diameter served as the substrate for the working electrode. A carbon rod and a saturated $Ag/AgCl/Cl^-$ were employed as the counter electrode and reference electrode, respectively. 5 mg of NiFe-PBA-gel-cal was dispersed in 4.5 mL of a water/isopropanol solution (1:3) containing 500 μL Nafion (5%). The resulting solution was sonicated for 0.5-1 h. When the solution was well dispersed, 4 μL of the above solution was dropped onto the clean GCE for electrochemical studies.

An O_2-purged aqueous solution of 1 M KOH (alkaline freshwater) and 1 M KOH and 0.5 M NaCl (alkaline simulated seawater) were the electrolytes for OER and HER experiments. Cyclic voltammetry (CV) curves were recorded at a sweep rate of 100 mV s^{-1} for multiple cycles. Linear sweep voltammetry (LSV) was carried out at a scan rate of 5 mV s^{-1} for polarization curves. LSV was performed several times until the signals were stabilized. The Tafel and EIS plot measurements were performed under the same conditions as for OER evaluation. CV curves with

different scan rates (10-60 mV s^{-1}) were measured over a potential range in which redox processes were absent to calculate the electrochemical double-layer capacitance: $C_{dl} = I_c/v$, where C_{dl}, I_c, and v are the double-layer capacitance (F cm^{-2}) of the electroactive materials, charging current (mA cm^{-2}), and scan rate (mV s^{-1}). All results reported in this work were converted to the RHE scale according to the Nernst equation without any *iR*-correction,

$$E_{RHE} = E_{Ag/AgCl/Cl^-} + 0.059 \times \text{pH} + E^0_{Ag/AgCl/Cl^-}$$

where $E_{Ag/AgCl/Cl^-}$ is the working potential, and $E^0_{Ag/AgCl/Cl^-}$ equals to 0.1976 V at 25 ^{o}C.

Turnover frequency (TOF) was calculated using the equation below,

$$\text{TOF} = (j \times \text{A}) / (4 \times \text{F} \times \text{n})$$

where j is the current density, A is the geometric area of electrode, F is the Faraday constant (96485 C mol^{-1}), and n is the moles of the corresponding metal atom (mol) within the catalyst loading.

A water-splitting device with a two-electrode configuration was assembled. Both the cathode and the anode electrodes were made by depositing NiFe-PBA-gel-cal onto Ni foam (2 × 1 cm^2) and then drying in air. To obtain a total catalyst loading of approximately 1 mg cm^{-2}, the deposition process was repeated several times. Then, the Ni foams loaded with catalyst were fixed as electrodes on both sides of an "H" tube with each component containing electrolyte, separated by a Nafion membrane.

Computational details

Density functional theory (DFT) calculation was performed using the generalized gradient approximation (GGA) Perdew-Burke-Ernzerhof (PBE) functional, and the projected augmented plane-wave method implemented in the Vienna *ab initio* simulation program (VASP) software code. A trigonal primitive unit cell containing two-unit formula of $Ni(OH)_2$ was used, and the plane-wave basis-set cutoff was set to 400 eV. The structure is of *P-3m1* space group, the same as our experimental XRD obtained data. The unit cell is *D3d* symmetry. The subsequent deprotonation modifications were carried out on the primitive unit cell. To obtain the projected density of state (PDOS), all structures were subjected to full relaxation until reaching a force threshold of 0.01 eV $Å^{-1}$. The Brillouin zone was sampled in a Monkhorst-Pack 3 × 3 × 2 k-points mesh for structural relaxation and 6 × 6 × 4 for static calculation. The Hubbard U correction was used to compensate the electron delocalization originated from GGA-PBE functional. The *Ueff*

was chosen to be 4 eV enforcing on Ni 3*d* atomic orbitals.

The Gibbs free energy of the adsorbed intermediate can be calculated as:

$$\Delta G = E_{ads} + \Delta E_{ZPE} - T\Delta S - \Delta G(\mathrm{pH}) + eU$$

where E_{ads} is the adsorption energy of intermediate, ΔE_{ZPE} is the zero-point energy difference between the adsorption state and gas state, T is the temperature (300 K), ΔS is the entropy various between the adsorption and gas phase.

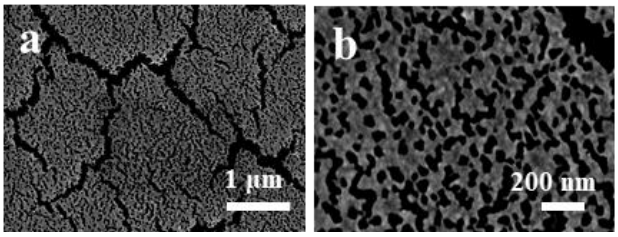

Figure S1. SEM images of FeFe-PBA-gel.

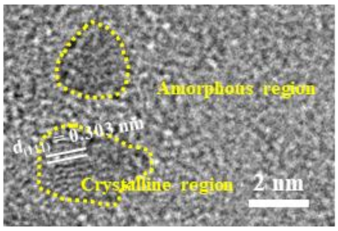

Figure S2. TEM image of the pseudo-amorphous NiC_x in NiFe-PBA-gel-cal.

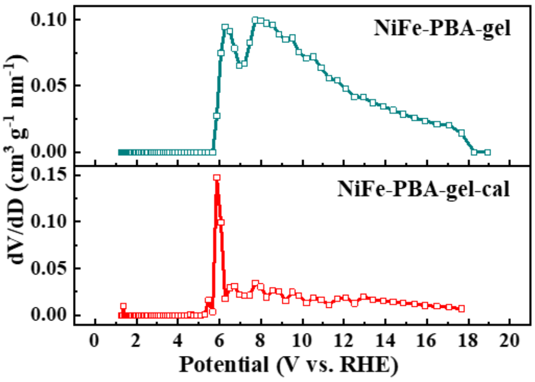

Figure S3. Pore distribution of NiFe-PBA-gel and NiFe-PBA-gel-cal.

Table S1. The Tafel slope and the overpotential comparison for NiFe-PBA-gel-cal and FeFe-PBA-gel-cal for electrochemical OER in both alkaline freshwater and alkaline simulated seawater.

**Catalyst**	**η_{100} OER (mV)**	**η_{500} OER (mV)**	**Tafel Slope (mV dec^{-1})**
**NiFe-PBA-gel-cal-F**	**308**	**398**	**63.1**
**NiFe-PBA-gel-cal-S**	**329**	**467**	**68.7**
**FeFe-PBA-gel-cal-F**	387	/	77.5
**FeFe-PBA-gel-cal-S**	435	/	83.9

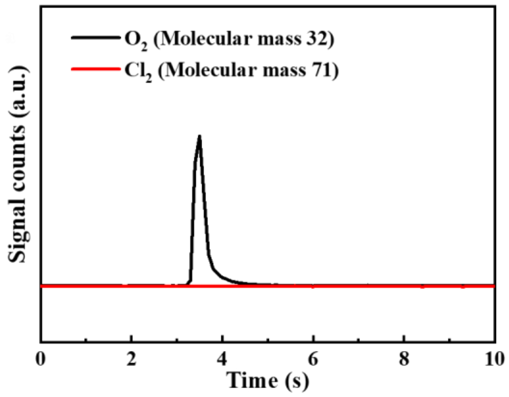

Figure S4. Time-series mass spectra of the produced gas during OER in alkaline simulated seawater.

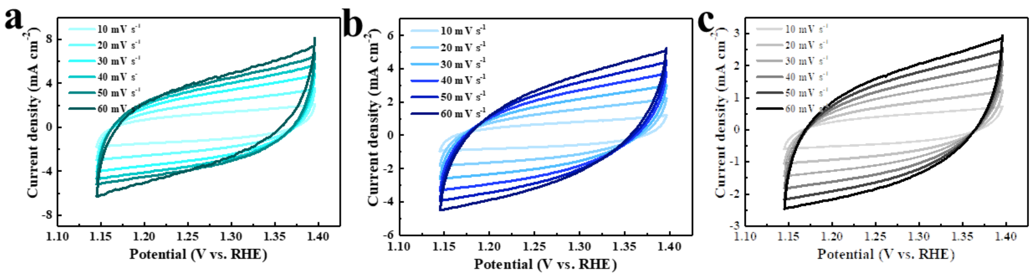

Figure S5. CV curves of (a) NiFe-PBA-gel-cal in alkaline simulated freshwater (b) FeFe-PBA-gel-cal in alkaline freshwater and (c) FeFe-PBA-gel-cal in alkaline simulated seawater at different scan rates.

Table S2. The value and error of each component in the equivalent circuit.

	R_s (Ω)	R_{ct} (Ω)	CPE-T	CPE-P	W_o-R	W_o-T	W_o-P
**NiFe-PBA-gel-cal-F**	2.17	13.59	8.52E-07	0.98	9.59	7.06E-03	0.41
**Error (%)**	1.06	0.36	3.24	0.29	4.93	6.96	1.50
**FeFe-PBA-gel-cal-F**	2.25	14.76	9.81E-07	0.97	5.17	6.76E-03	0.31
**Error (%)**	1.18	0.37	3.54	0.32	1.69	3.01	1.51
**NiFe-PBA-gel-cal-S**	2.36	22.15	9.03E-07	0.92	26.84	7.36E-04	0.47
**Error (%)**	6.69	1.62	6.65	1.23	2.33	3.09	1.74

Table S3. OER activity comparison among NiFe-PBA-gel-cal catalyst and other reported NiFe-based non-noble metal electrocatalysts in alkaline freshwater (1 M KOH) at room temperature.

Catalyst	Substrate	η_{100} (mV vs. RHE)	η_{500} (mV vs. RHE)	Ref.
**NiFe-PBA-gel-cal**	**Glassy carbon**	**281**	**466**	**This work**
NiCoFe-MOF	Ni foam	310	NA	*Adv. Mater.* **2019**, *31*, 1901139
FeNiP/NCH	Glassy carbon	340	NA	*J. Am. Chem. Soc.* **2019**, *141*, 7906-7916
$Mn_{0.5}(Fe_{0.3}Ni_{0.7})_{0.5}O_x$	Glassy carbon	430	NA	*Adv. Funct. Mater.* **2020**, *30*, 1905992
NiFe LDH/graphene	Glassy carbon	325	NA	*Adv. Mater.* **2017**, *29*, 1700017
NiFe LDH	Ni foam	450	NA	*Science* **2014**, *345*, 1593-1596

Table S4. OER activity comparison among NiFe-PBA-gel-cal catalyst and other reported Ni-based non-noble metal electrocatalysts in alkaline simulated seawater and neutral electrolytes at room temperature.

Catalyst	Electrolyte	Current density (mA cm^{-2})	Overpotential (mV)	Ref.
**NiFe-PBA-gel-cal**	**1 M KOH + 0.5 M NaCl**	**100**	**329**	**This work**
NiFe LDH	0.1 M KOH + 0.5 M NaCl	10	359	*ChemSusChem* **2016**, *9*, 962-972
NiFe/NiS$_x$-Ni	1 M KOH + 0.5 M NaCl	100	286	*Proc. Natl. Acad. Sci. U. S. A.* **2019**, *116*, 6624-6629
Ni$_2$P-Fe$_2$P	1 M KOH + 0.5 M NaCl	100	~334	*Adv. Funct. Mater.* **2021**, *31*, 2006484.
NiCo-DEA	1 M KOH+ natural seawater	100	~ 670	*Electrochim. Acta* **2017**, *247*, 381-391
NiP$_2$/CC	Natural seawater	4	581	*New J. Chem.* **2017**, *41*, 2154-2159.

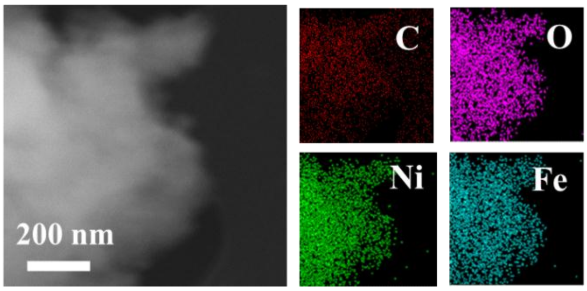

Figure S6. HAADF-STEM image and EDS-mapping images of NiFe-PBA-gel-cal after OER.

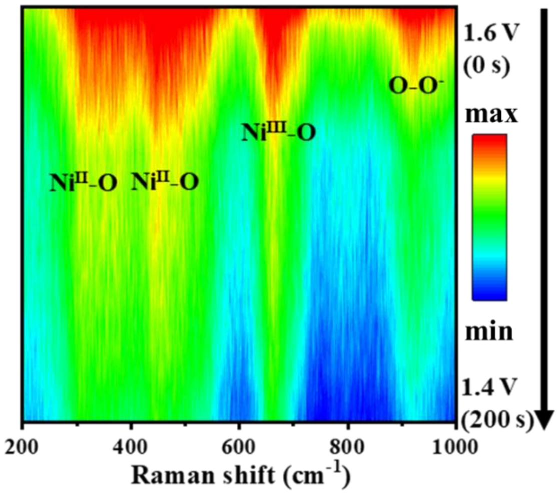

Figure S7. *Operando* Raman spectra contour plot of NiFe-PBA-gel-cal obtained from the voltage decreasing from 1.6 to 1.4 V (vs. RHE).

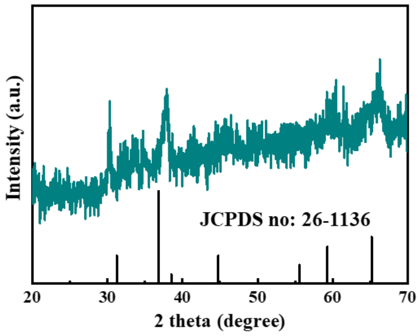

Figure S8. RD pattern of NiFe-PBA-gel-cal after OER.

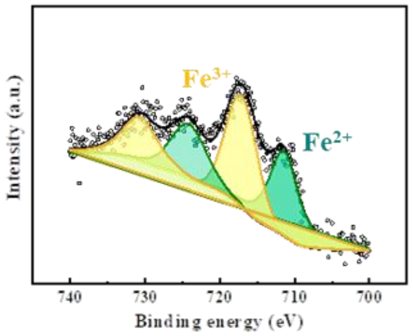

Figure S9. XPS high-resolution spectra at Fe 2*p* of NiFe-PBA-gel-cal after OER.

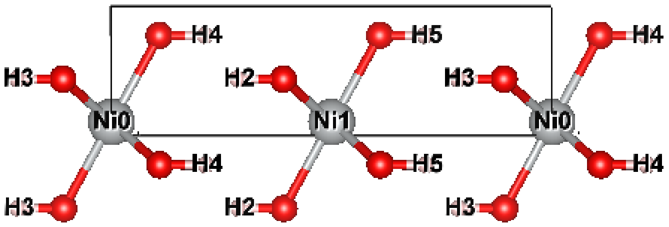

Figure S10. Side view of primitive unit cell of $Ni(OH)_2$. Two unit formula of $Ni(OH)_2$ are presented in the periodic boundary condition (PBE) unit cell. The two Ni atoms are marked as Ni0 and Ni1 with their subsequent OH groups attached.

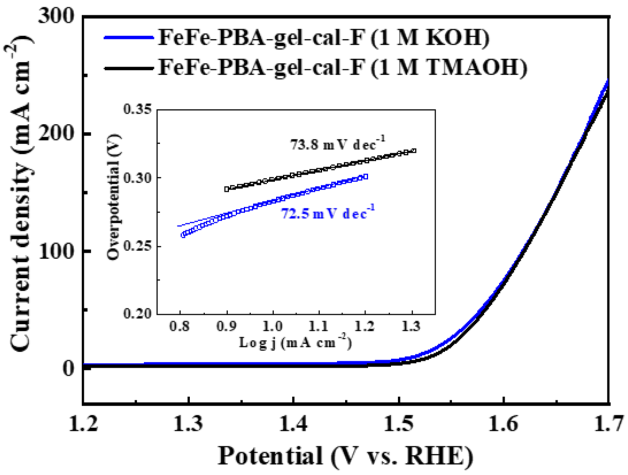

Figure S11. LSV curves of FeFe-PBA-gel-cal in 1 M KOH (blue) and 1 M TMAOH (black). Inset shows the corresponding Tafel plots.

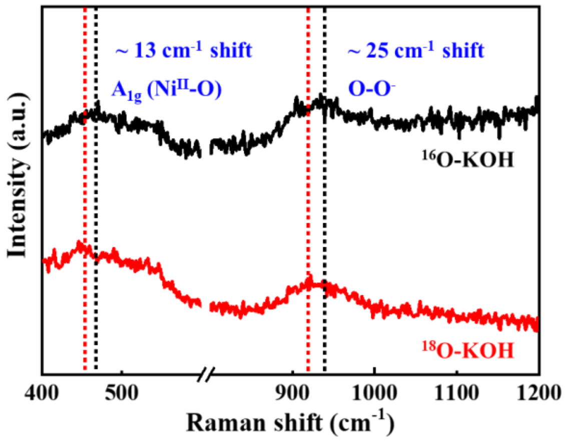

Figure S12. *Operando* Raman spectra of $H_2^{16}O$-activated NiFe-PBA-gel-cal performing OER in $H_2^{16}O$ (black) and $H_2^{16}O$ (red), respectively, the spectra were recorded at 1.6 V vs. RHE. The peak of the A_{1g} mode of Ni^{II}-O shifted by ~13 cm^{-1}, while the peak of the superoxo-like (OO^-) species at the higher wavenumbers shifted by ~25 cm^{-1}.

Table S5. The Tafel slope and the overpotential comparison of NiFe-PBA-gel-cal for electrochemical HER in alkaline freshwater and simulated seawater.

Catalyst	η_{100} HER (mV)	η_{500} HER (mV)	Tafel Slope (mV dec^{-1})
**NiFe-PBA-gel-cal-F**	**281**	**466**	**82.4**
**NiFe-PBA-gel-cal-S**	480	/	160.8

Table S6. HER activity comparison among NiFe-PBA-gel-cal catalyst and other reported Ni-based non-noble metal electrocatalysts in alkaline freshwater (1 M KOH) at room temperature.

Catalyst	Substrate	η_{100} (mV vs. RHE)	η_{500} (mV vs. RHE)	Ref.
**NiFe-PBA-gel-cal**	**Glassy carbon**	**308**	**398**	**This work**
Ni@NC	Glassy carbon	~310	NA	*Adv. Mater.* **2017**, *29*, 1605957
Co_xNi_yP NTs	Glassy carbon	~490	NA	*Adv. Funct. Mater.* **2017**, *27*, 1703455
Ni_5P_4	Glassy carbon	~323	NA	*Angew. Chem. Int. Ed.* **2015**, *54*, 12361-12365
$NiCo_2S_4$ nanowire	Glassy carbon	~350	NA	*Adv. Funct. Mater.* **2016**, *26*, 4667-4677
Ni_3S_2	Ni foam	~400	NA	*J. Am. Chem. Soc.* **2015**, *137*, 14023-14026

Table S7. Overall freshwater splitting activity comparison among NiFe-PBA-gel-cal catalyst and other reported NiFe-based non-noble metal electrocatalysts in alkaline freshwater (1 M KOH) at room temperature.

Catalyst	Substrate	Current density (mA cm^{-2})	Voltage (V)	Ref.
**NiFe-PBA-gel-cal**	**Ni Foam**	**100**	**1.57**	**this work**
NiFeRu LDH	Ni Foam	100	1.705	*Adv. Mater.* **2018**, *30*, 1706279
$(Ni_{0.33}Fe_{0.67})_2P$	Ni Foam	100	1.72	*Adv. Funct. Mater.* **2017**, *27*, 1702513
Ni-ZIF/Fe-B	Ni Foam	100	1.78	*Adv. Energy Mater.* **2020**, *10*, 1902714
$Fe_{0.09}Co_{0.13}$-$NiSe_2$	Carbon Fiber Cloth	100	1.69	*Adv. Mater.* **2018**, *30*, 1802121
FeCoNi-HNTAs	Ni Foam	100	1.725	*Nat. Commun.* **2018**, *9*, 2452

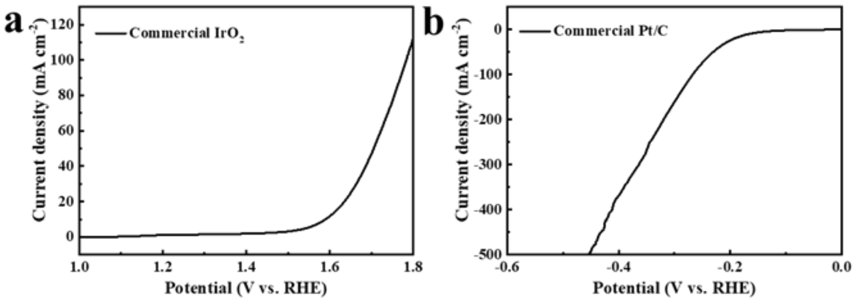

Figure S13. The OER performance of commercial IrO_2 and the HER performance of commercial Pt/C (10% wt. Pt).

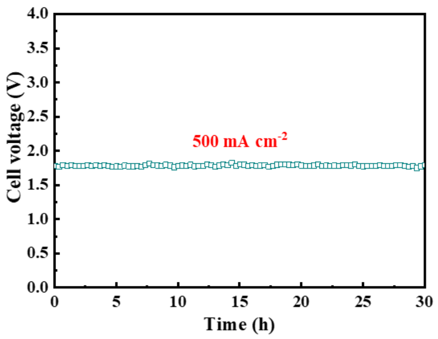

Figure S14. Chronopotentiometry stability test for NiFe-PBA-gel-cal//NiFe-PBA-gel-cal electrode couple at a current density of 500 mA cm^{-2}.

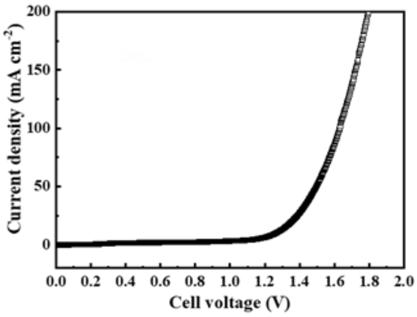

Figure S15. Overall water splitting performance of NiFe-PBA-gel-cal//NiFe-PBA-gel-cal electrode couple in alkaline real seawater.

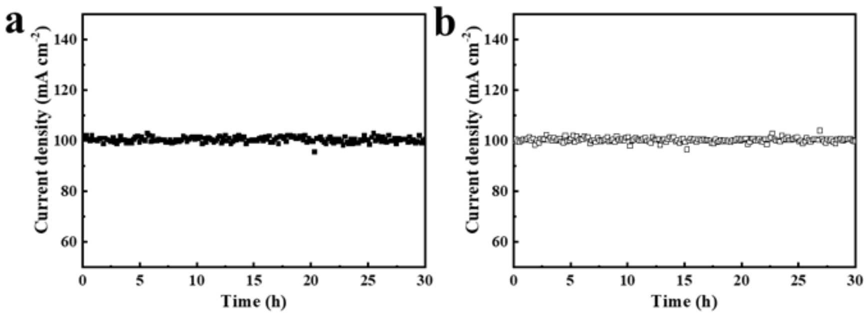

Figure S16. Chronoamperometry stability test for NiFe-PBA-gel-cal//NiFe-PBA-gel-cal electrode couple in (a) alkaline real seawater and (b) alkaline simulated seawater at voltages of 1.64 and 1.66 V, respectively.

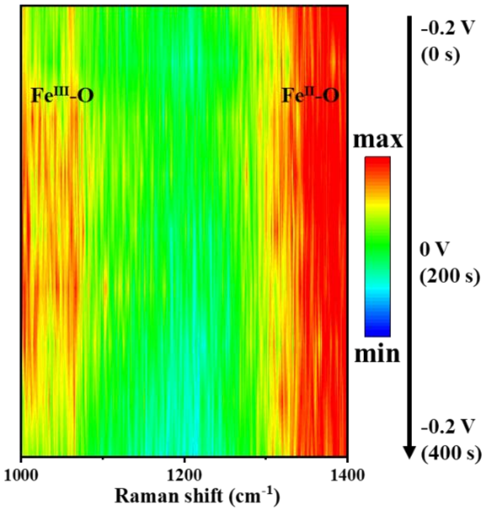

Figure S17. Normalized *Operando* Raman spectra contour plot of NiFe-PBA-gel-cal obtained from the voltage increasing from -0.2 to 0 to -0.2 V (vs RHE).

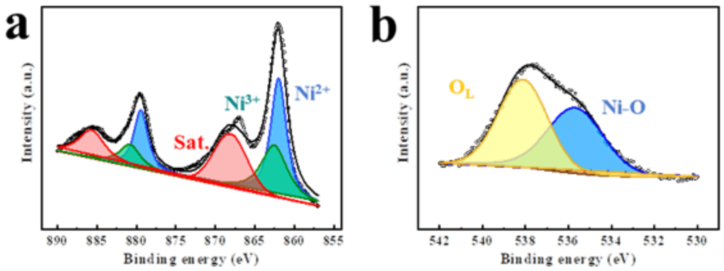

Figure S18. XPS high-resolution spectra at (a) Ni 2*p* and (b) O 1*s* of NiFe-PBA-gel-cal after HER.

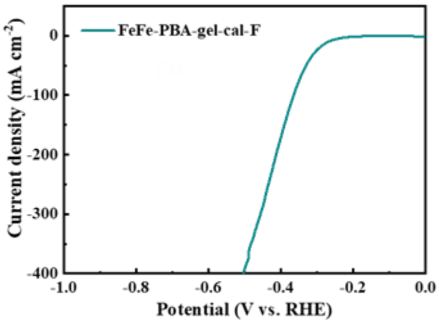

Figure S19. The HER performance of FeFe-PBA-gel-cal in alkaline freshwater.
